# Parental transmission of smoking among middle-aged and older populations in Russia and Belarus

**DOI:** 10.1007/s00038-017-1068-0

**Published:** 2018-01-05

**Authors:** Alexi Gugushvili, Martin McKee, Aytalina Azarova, Michael Murphy, Darja Irdam, Lawrence King

**Affiliations:** 1 Department of Social Policy and Intervention and Nuffield College, University of Oxford, Barnett House, 32 Wellington Square, Oxford, OX1 2ER UK; 2 Department of Sociology, University of Cambridge, Cambridge, UK; 3 London School of Hygiene and Tropical Medicine, London, UK; 4 London School of Economics and Political Science, London, UK

**Keywords:** Intergenerational transmission, Smoking, Demographic cohort study, Russia, Belarus, Multilevel Poisson analysis

## Abstract

**Objectives:**

The very high rates of smoking among men and the rapid changes among women in the Post-Soviet countries mean that this region offers an opportunity to understand better the intergenerational role of parental influences on smoking.

**Methods:**

In this study, we exploit a unique data set, the PrivMort cohort study conducted in 30 Russian and 20 Belarusian towns in 2014–2015, which collects information on behaviours of middle-aged and older individuals and their parents, including smoking. We explored the associations between smoking by parents and their offspring using multiply imputed data sets and multilevel mixed-effect Poisson regressions.

**Results:**

Adjusting for a wide array of social origin, socio-demographic, and socio-economic variables, our analysis suggests that sons of regularly smoking fathers have prevalence ratios of 1.35 [95% confidence intervals (CI) 1.21–1.50] and 1.39 (CI 1.23–1.58) of smoking, while the figures for daughters of regularly smoking mothers are 1.91 (CI 1.40–2.61) and 2.30 (CI 1.61–3.28), respectively, in Russia and Belarus.

**Conclusions:**

Intergenerational paternal and maternal influences on smoking should be taken into account in studies seeking to monitor the rates of smoking and the impact of tobacco control programmes.

## Introduction

According to the World Health Organization’s (WHO) estimates, post-Soviet countries, including Russia and Belarus, have some of the highest prevalence of smoking among adult men anywhere in the world, while rates have been increasing rapidly among women in the past 2 decades (McKee et al. 1998; Perlman et al. 2007; Roberts et al. 2012; Quirmbach and Gerry 2016; WHO 2016). The previous research has studied the macro-level effects of economic transition and market entry of transnational tobacco companies on these patterns (Gilmore and McKee 2005; Gilmore et al. 2011; Lillard and Dorofeeva 2015), as well as individual-level factors such as age, marital status, family disruption, education, urban residency, household economic situation, self-reported material deprivation, unemployment, occupation, and religious denomination (Gilmore et al. 2001; Pomerleau et al. 2004; Bobak et al. 2006; Perlman et al. 2007; Kislitsyna et al. 2010; Roberts et al. 2012). One area that has largely been overlooked in this research has been intergenerational transmission of propensity to smoke from parents to their offspring in the post-Soviet context.

Studies on social determinants of smoking have repeatedly demonstrated intergenerational transmission of smoking in various settings, populations, and times (Bailey et al. 1993; McGee et al. 2006; Brook et al. 2013; Christopoulou et al. 2013; Kandel et al. 2015). Nonetheless, one major limitation of this literature is that it is almost exclusively concerned with intergenerational transmission of smoking to adolescents and individuals in their 20s and 30s, while the role of parental smoking later in life is under-researched. The available research also suggests that, if they are to identify the net intergenerational transmission of smoking, empirical studies should adequately account for socio-economic status in each generation, which independently affects tobacco use (Conrad et al. 1992; Jefferis et al. 2003; Schori et al. 2014). Lower parental education and material deprivation in childhood, for instance, predicts an individuals’ increased risk of smoking in their adulthood and may explain spurious evidence of an intergenerational transmission of smoking (Fagan et al. 2005). Few existing studies account for social origin variables separately for fathers and mothers and so might overestimate the effect of parental smoking on their children’s smoking propensity.

The very high rates of smoking among men and the rapid changes among women in Russia and Belarus mean that these countries offer an opportunity to understand better the intergenerational role of parental influences on smoking. We are aware of only two studies in post-Soviet settings that investigate the link between parents and their offspring smoking (Kemppainen et al. 2006; Kislitsyna et al. 2010). These are limited in that they only include adolescents, cover limited geographical areas with small samples, do not account for effects with social origins, such as fathers’ and mothers’ education, and do not consider a wide array of confounding factors in an individual’s life, such as labour market characteristics, that might explain intergenerational transmission of smoking between parents and their children. The goal of the study is to exploit a unique data set from Russia and Belarus that can address many of the limitations of previous research by examining the association between fathers’ and mothers’ smoking and smoking in their middle-aged and older offspring, after taking account of those offspring’s social origin, socio-demographic, and socio-economic characteristics.

## Methods

### Data set

Our analysis is based on the PrivMort data set, collected in 2014–2015 within a multi-disciplinary project whose main objective is to investigate the post-socialist morbidity and mortality crisis by means of a cross-sectional retrospective cohort study. Initially, the PrivMort collected basic economic, demographic, and enterprise-level data on all towns with 10,000–100,000 inhabitants in the European part of Russia and in Belarus, excluding the regions of the North Caucasus. A set of 30 and 20 towns was selected from the pool of 539 and 96, respectively, in Russia and Belarus, using the method of propensity score matching based on the following pre-transition demographic and socio-economic characteristics of the towns: (1) crude death rates per 1000 population; (2) population size, (3) dependency ratio; (4) average wage in US dollars; (5) number of physicians per 10,000 population; (6) floor area per person; (7) death rates from alcohol poisoning per 100,000 population; and (8) emission of pollutants into the atmosphere from stationary sources, thousand tons.

In the selected towns, a random walk procedure was used for sampling the respondents. The towns were divided into street-centred clusters, which were then distributed among the interviewers using the method of random numbers. Interviewers conducted face-to-face interviews using structured questionnaires. Response rate was higher in Russia (48%) than in Belarus (39%). Full details concerning the selection of towns and other aspects of the PrivMort methodology are given elsewhere (Irdam et al. 2016; Azarova et al. 2017; Gugushvili et al. 2018).

To be included in the survey, a potential respondent had to be born before 1972. This criterion ensured that a respondent had reached working age by 1991. The respondent sample, therefore, includes only those aged 42 and over. For robustness of analysis, we further censor our sample to working age individuals 65 and younger. In addition to information collected on respondents’ smoking, socio-demographic, and socio-economic characteristics, the PrivMort survey collected data on their fathers’ and mothers’ characteristics, including their smoking patterns, and educational attainment. The actual data set that we employ is one derived from a multiple imputation exercise via the MICE (Multiple Imputation using Chained Equations) package in Stata 14, allowing for 20 sets of multiple imputations and combining them using Rubin’s (1987) rules. We undertake the latter procedure to compensate for the extent of missing data in our key variables—paternal and maternal smoking. Overall, our analytical samples consist of 15,098 individuals interviewed in Russia and 10,370 in Belarus. Although we stratify our analysis by gender, in both countries, women are overrepresented in the data set (70.3 and 71.6%, respectively).

### Statistical analysis

To understand the patterns of intergenerational transmission of smoking among men and women, accounting for various individual-level covariates described in “Results” section, we create a dummy variable for regular smokers that takes value of 1 if they smoked at the time of interview and zero otherwise. We consecutively fit age-adjusted bivariate and multivariate multilevel mixed-effect Poisson regressions with robust variance separately by gender. In the latter models, level 1 consists of individuals and level 2 consists of towns in which the PrivMort survey was conducted. For the latter level, we account for the size of population (the mean value is 29,885 in Russia and 47,556 in Belarus). Models are estimated using Stata 14 function “mepoisson” and the results are presented as prevalence ratios (OR) with corresponding 95% confidence intervals (CI). In addition to reporting relative measures of intergenerational transmission of smoking, we also calculate post-estimation predicted probabilities for men and women with varying patterns of parental smoking averaged across the relevant populations in Russia and Belarus.

## Results

### The prevalence of smoking

The PrivMort survey asked respondents if they smoked. The available response options were: (1) never smoked, (2) used to smoke but quit, and (3) currently regular smoker. Table 1 shows the prevalence of smoking in Russia and Belarus. More than a half of male respondents in both countries are current smokers. The prevalence of current smoking among women is just under 10% in both countries. The latter is lower rate than those reported in the above-mentioned nationally representative surveys of adult populations (14.4% for Russia and 13.2% for Belarus).

**Table 1:** Prevalence of smoking among respondents and their parents, percent (PrivMort retrospective cohort study conducted in Russia and Belarus, 2014–2015)

	Men				Women			
	Russia		Belarus		Russia		Belarus	
	Sons	Fathers	Sons	Fathers	Daughters	Mothers	Daughters	Mothers
Never	25.0	22.6	31.3	28.3	84.7	96.6	87.7	96.7
Regular smoker	53.3	58.5	51.9	53.6	9.4	1.6	8.3	2.0
Quit	21.8	18.9	16.7	18.1	5.9	1.8	4.0	1.3

Table 1 also shows the prevalence of smoking among the sample of fathers and mothers in Russia and Belarus. Respondents reported their parents’ current smoking behaviour if they were alive at the time of interview, and for deceased parents information on smoking characteristics was collected before their death. Since answer options for parents, as for respondents, include ‘used to smoke but quit’, we are able obtain a good approximation of smoking histories of all parents. By comparing the shares of regular smokers between respondents’ and parental generations, we see that, among men, the prevalence of smoking decreased by about 5 and 2 percentage points, respectively, in Russia and Belarus. Among mothers, the share of those who smoked regularly or smoked and ceased was marginal, at 1.6–2.0 and 1.8–1.3% in considered countries. In both countries, we observe that fathers are less likely to be never smokers than sons, while mothers are more likely to be never smokers than daughters.

### Covariates

Table 2 presents the frequencies of covariates in our sample that are used in the multivariate analysis of the intergenerational role of parental influences on smoking. The descriptive statistics suggest that, for most variables, Belarus and Russia are quite similar. We classify parents’ and respondents’ educational attainment in primary, secondary, and tertiary education. Respondents have significantly higher qualifications than their fathers and mothers. In addition, we control for childhood deprivation reported by respondents. The share of individuals stating that they often or constantly went to bed hungry when they were children is about 10% among men and 7–8% among women. We categorise respondents’ age into five groups (42–45, 46–50, 51–55, 56–60, and 61–65) and use this variable to estimate age-adjusted prevalence ratios. Marital status is categorised as single, married, separated/divorced, and widowed. About three-fifths of men are in work, while women are roughly equally distributed between working and not working groups. In both countries, more men than women have experienced long-term unemployment at least once in their lives by looking for work continuously for 6 months. The share of men who have attained supervisory status in their employment is slightly higher than the share of women. Finally, we also account for respondents’ religious denomination.

**Table 2:** Descriptive statistics of covariates of regular smoking, percent (PrivMort retrospective cohort study conducted in Russia and Belarus, 2014–2015)

	Men		Women	
	Russia	Belarus	Russia	Belarus
Father’s education				
Primary	59.5	50.5	64.3	55.1
Secondary	34.8	23.2	30.7	20.5
Tertiary	5.7	26.2	5.0	24.4
Mother’s education				
Primary	60.8	51.7	66.1	59.0
Secondary	32.4	18.8	29.4	16.0
Tertiary	6.8	29.5	4.5	25.0
Childhood deprivation				
Never	87.9	91.5	87.9	92.2
Occasionally went to bed hungry	9.9	7.6	10.0	6.7
Often went to bed hungry	1.6	0.5	1.3	0.7
Constantly hungry	0.6	0.3	0.8	0.4
Age group				
42–45	24.6	14.6	18.5	9.9
46–50	18.4	16.6	15.8	14.1
51–55	16.0	17.5	15.2	16.7
56–60	19.3	23.3	21.3	26.0
61–65	21.7	27.9	29.2	33.3
Marital status				
Single	8.7	6.7	5.0	2.9
Married	69.9	68.6	59.5	59.7
Separated/divorced	16.1	20.0	15.5	15.5
Widow/widower	5.3	4.7	20.0	21.9
Education				
Primary	29.0	25.3	25.6	20.9
Secondary	53.1	57.4	54.6	61.4
Tertiary	17.9	17.4	19.8	17.8
Employment				
Not working	38.2	38.5	46.7	53.9
Working	61.8	61.5	53.3	46.1
Long-term unemployment				
No	77.4	85.8	82.1	90.7
Yes	22.6	14.2	17.9	9.3
Supervisory status				
No	77.5	78.8	80.0	80.2
Yes	22.5	21.2	20.0	19.8
Religion				
Orthodox	89.1	83.6	94.3	85.8
Other Christian	1.2	9.9	1.3	12.6
Muslim	1.2	0.2	1.3	0.1
Other	8.5	6.4	3.0	1.5
Observations	4517	3029	10,581	7341

### Bivariate analysis

In Table 3, we present age-adjusted bivariate prevalence ratios of regular smoking in Russia and Belarus. We observe that father’s regular smoking and smoking cessation are positively associated with their son’s propensity to smoke. Having regularly smoking fathers is also linked to their daughters’ smoking, but the prevalence ratio in Belarus is lower than that observed for sons in this country. On the other hand, mother’s smoking is associated with 2.2 and 2.7 times higher prevalence that their daughters are current smokers in Russia and Belarus. The latter association is not statistically significant among sons. It is also noticeable that the town-level variance in smoking is much higher for women that for men.

**Table 3:** Age-adjusted bivariate prevalence ratios of regular smoking from multilevel mixed-effect Poisson regressions (PrivMort retrospective cohort study conducted in Russia and Belarus, 2014–2015)

	Men		Women	
	Russia PR [95 CI]	Belarus PR [95 CI]	Russia PR [95 CI]	Belarus PR [95 CI]
Intercept	0.39 [0.35, 0.42]	0.36 [0.30, 0.42]	0.03 [0.02, 0.04]	0.02 [0.01, 0.03]
Father’s smoking				
Never smoked	1.00	1.00	1.00	1.00
*N*	987	804	2425	2127
Regular smoker	1.38 [1.24, 1.53]	1.45 [1.29, 1.64]	1.40 [1.19, 1.66]	1.36 [1.08, 1.72]
*N*	2610	1623	6221	3937
Quit	1.11 [0.99, 1.24]	1.28 [1.13, 1.44]	0.93 [0.73, 1.19]	1.20 [0.95, 1.51]
*N*	920	602	1935	1.313
Mother’s smoking				
Never smoked	1.00	1.00	1.00	1.00
*N*	4351	2901	10,236	7129
Regular smoker	1.16 [0.96, 1.40]	1.13 [0.88, 1.45]	2.16 [1.60, 2.91]	2.66 [1.95, 3.63]
*N*	76	69	170	139
Quit	1.00 [0.79, 1.27]	0.87 [0.68, 1.12]	1.92 [1.33, 2.79]	1.64 [1.12, 2.39]
*N*	90	60	175	73
Random intercept	0.01 [0.00, 0.02]	0.01 [0.00, 0.04]	0.28 [0.12, 0.64]	0.13 [0.05, 0.32]
Model statistics				
Towns	30	20	30	20
Observations	4517	3029	10,581	7341

### Multivariate analysis

In Table 4, prevalence ratios from multilevel mixed-effect Poisson regressions suggest that smoking patterns of parents are, indeed, associated with individuals’ propensity to smoke after adjustment for all covariates. Significant differences between genders are also apparent. Father’s smoking is a significant predictor of regular smoking among men with prevalence ratios of 1.35 (CI 1.21–1.50) and 1.39 (CI 1.23–1.58), respectively, in Russia and Belarus. The latter associations among daughters are also significant in both countries with prevalence ratios of 1.40 (CI 1.17–1.66) and 1.33 (CI 1.04–1.69). Father’s experience of quitting smoking is positively associated with regular smoking among men but not among women. For the association between mothers and their children, statistically significant prevalence ratios are observed among daughters but not among sons. Having smoking mothers is associated with 1.91 (CI 1.40–2.61) and 2.30 (CI 1.61–3.28) times higher prevalence of being regular smoker among daughters, respectively, in Russia and Belarus. Furthermore, having mothers who quit smoking is associated with 1.83 (CI 1.23–2.72) times higher prevalence of regular smoking in Russia.

**Table 4:** Age-adjusted multivariate prevalence ratios of regular smoking from multilevel mixed-effect Poisson regressions (PrivMort retrospective cohort study conducted in Russia and Belarus, 2014–2015)

	Men		Women	
	Russia PR [95 CI]	Belarus PR [95 CI]	Russia PR [95 CI]	Belarus PR [95 CI]
Fixed effects				
Intercept	0.27 [0.21, 0.35]	0.28 [0.25, 0.31]	0.02 [0.01, 0.03]	0.01 [0.01, 0.01]
Parental and childhood variables				
Father’s smoking				
Never	1.00	1.00	1.00	1.00
Regular smoker	1.35 [1.21, 1.50]	1.39 [1.23, 1.58]	1.40 [1.17, 1.66]	1.33 [1.04, 1.69]
Quit	1.11 [0.99, 1.24]	1.23 [1.10, 1.38]	0.95 [0.74, 1.21]	1.21 [0.97, 1.52]
Mother’s smoking				
Never	1.00	1.00	1.00	1.00
Regular smoker	1.13 [0.93, 1.36]	1.05 [0.84, 1.33]	1.91 [1.40, 2.61]	2.30 [1.61, 3.28]
Quit	0.97 [0.77, 1.22]	0.88 [0.68, 1.15]	1.83 [1.23, 2.72]	1.44 [0.93, 2.21]
Father’s education				
Primary	1.00 [0.82, 1.22]	0.93 [0.82, 1.04]	0.65 [0.51, 0.84]	0.99 [0.77, 1.27]
Secondary	1.05 [0.89, 1.24]	0.93 [0.83, 1.04]	0.78 [0.59, 1.02]	0.93 [0.72, 1.21]
Tertiary	1.00	1.00	1.00	1.00
Mother’s education				
Primary	1.06 [0.91, 1.24]	1.05 [0.95, 1.15]	0.97 [0.69, 1.38]	0.71 [0.56, 0.91]
Secondary	1.14 [0.99, 1.31]	1.03 [0.94, 1.13]	1.15 [0.82, 1.62]	0.96 [0.81, 1.13]
Tertiary	1.00	1.00	1.00	1.00
Childhood deprivation				
Never	1.00	1.00	1.00	1.00
Occasionally went to bed hungry	1.00 [0.88, 1.14]	0.92 [0.79, 1.06]	1.19 [0.97, 1.47]	1.29 [1.06, 1.56]
Often went to bed hungry	1.21 [1.01, 1.45]	0.79 [0.45, 1.38]	1.61 [0.98, 2.63]	1.17 [0.45, 3.04]
Constantly hungry	1.19 [0.80, 1.79]	1.25 [0.79, 2.00]	1.52 [0.91, 2.55]	2.62 [1.13, 6.06]
Respondents’ characteristics				
Marital status				
Single	0.91 [0.79, 1.05]	1.05 [0.94, 1.17]	1.74 [1.35, 2.25]	1.87 [1.26, 2.78]
Married	1.00	1.00	1.00	1.00
Separated/divorced	1.07 [0.98, 1.17]	1.21 [1.11, 1.33]	1.74 [1.47, 2.06]	1.84 [1.53, 2.22]
Widow/widower	1.09 [0.95, 1.24]	1.09 [0.95, 1.25]	1.76 [1.53, 2.04]	1.71 [1.40, 2.08]
Education				
Primary	1.45 [1.27, 1.66]	1.49 [1.36, 1.62]	1.93 [1.49, 2.51]	2.02 [1.55, 2.64]
Secondary	1.38 [1.21, 1.56]	1.41 [1.30, 1.53]	1.39 [1.08, 1.79]	1.59 [1.36, 1.87]
Tertiary	1.00	1.00	1.00	1.00
Employment				
Not working	1.00	1.00	1.00	1.00
Working	0.95 [0.89, 1.02]	0.92 [0.83, 1.01]	0.95 [0.75, 1.20]	0.74 [0.60, 0.91]
Long-term unemployment				
No	1.00	1.00	1.00	1.00
Yes	1.09 [1.00, 1.18]	1.12 [1.01, 1.24]	1.52 [1.28, 1.82]	1.49 [1.17, 1.91]
Supervisory status				
No	1.00	1.00	1.00	1.00
Yes	0.90 [0.83, 0.98]	0.95 [0.88, 1.03]	0.92 [0.77, 1.11]	1.09 [0.90, 1.31]
Religion				
Orthodox	1.00	1.00	1.00	1.00
Other Christian	0.69 [0.45, 1.05]	0.88 [0.77, 1.01]	1.10 [0.73, 1.65]	1.08 [0.87, 1.34]
Muslim	1.01 [0.81, 1.27]	0.81 [0.46, 1.41]	1.29 [0.83, 2.01]	0.91 [0.49, 1.72]
Other	1.09 [1.00, 1.19]	0.90 [0.79, 1.03]	1.28 [0.97, 1.71]	0.95 [0.64, 1.41]
Macro-level variable				
Size of town (standardized)	0.98 [0.94, 1.01]	0.94 [0.90, 0.98]	1.03 [0.88, 1.21]	1.23 [0.99, 1.52]
Random intercept	0.01 [0.00, 0.03]	0.01 [0.00, 0.13]	0.25 [0.11, 0.55]	
Model statistics				
Towns	30	20	30	20
Observations	4517	3029	10,581	7341

Once parental smoking behaviour is accounted for, parental educational attainment is not significantly linked to offspring’s likelihood of smoking. However, compared to having tertiary educated fathers, women with low educated fathers in Russia are less likely to be regular smokers. Among women in Belarus, we also see that the daughters of low educated mothers in comparison to tertiary educated mothers have 29% lower prevalence of smoking. We do not find that childhood deprivation is systematically and significantly related to smoking. However, Russian men that often went to bed hungry, and Belarusian women who occasionally and constantly went to bed hungry when they were children, are more likely to be regular smokers.

When examined by marital status, single and widowed women and separated/divorced men and women have significantly higher prevalence of smoking than married individuals. Respondents’ own educational attainment is a strong predictor of regular smoking in both countries and across gender. For instance, men with primary education have prevalence ratios of 1.45 (CI 1.27–1.66) and 1.49 (CI 1.36–1.62) of being regular smokers in Russia and Belarus compared with those with tertiary education. Turning to labour market characteristics, being in employment, is associated with lower prevalence of smoking among women in Belarus, while long-term unemployment significantly increases the chances of regular smoking with prevalence ratios of 1.09 (CI 1.00–1.18) and 1.12 (CI 1.01–1.24) for men in Russia and Belarus and prevalence ratios of 1.52 (CI 1.28–1.82) and 1.49 (CI 1.17–1.91) for women in the same countries. Men with supervisory status attainment are also less likely to smoke in Russia. Neither respondent’s religious denomination nor the size of population in towns where the PrivMort retrospective cohort study was conducted is significantly and systematically related to regular smoking.

**Fig. 1:**
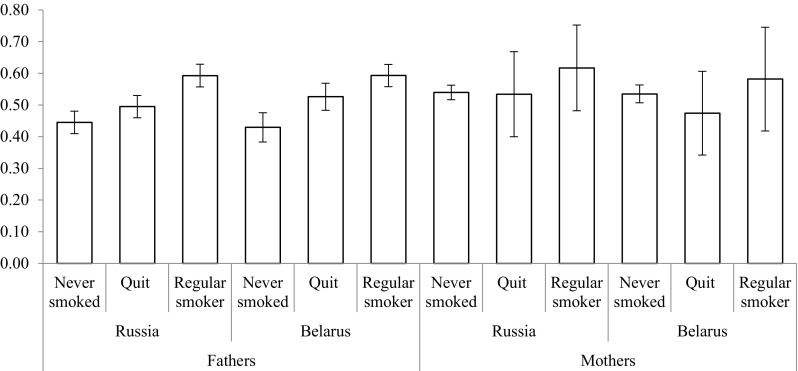
Predicted probabilities of regular smoking among men conditioned by parental smoking (PrivMort retrospective cohort study conducted in Russia and Belarus, 2014–2015). Error bars represent 95% confidence intervals

**Fig. 2:**
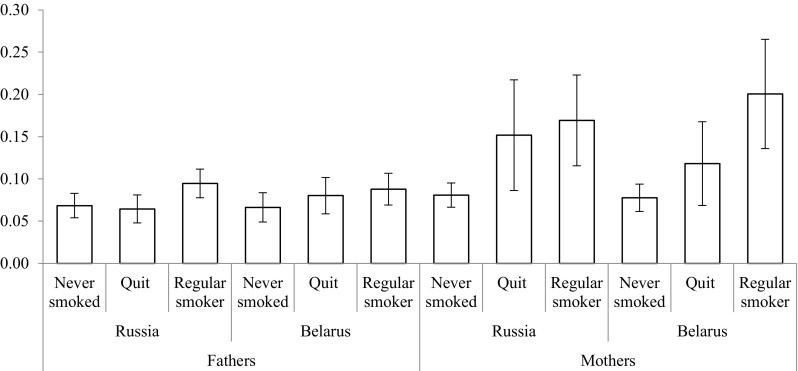
Predicted probabilities of regular smoking among women conditioned by parental smoking (PrivMort retrospective cohort study conducted in Russia and Belarus, 2014–2015). Error bars represent 95% confidence intervals

To show the overall differences in smoking at the population level conditioned by parental smoking patterns, we calculate predicted probabilities. In Fig. 1, we see that accounting for other social background and own socio-demographic and socio-economic variables, as shown in Table 4, men whose father never smoked have a 0.45 (CI 0.41–0.48) probability of being regular smokers in Russia, whereas for men with regularly smoking father, this probability is 14 percentage points higher (0.59 CI 0.56–0.63). This association is even more pronounced in Belarus where having a regularly smoking father is associated with up to 16 percentage points higher probability of being a regular smoker when compared to having a father that never smoked. Figure 2 indicates that women in Russia with never smoking mothers have a probability of 0.08 (CI 0.07–0.10) of being regular smokers against the probability of 0.17 (CI 0.12–0.22) for having regularly smoking mothers. In Belarus, the size of this association is even larger; having smoking mothers is associated with a 12.3 percentage points higher probability of smoking.

### Further analysis

In unreported analysis, we have also conducted additional tests. To check if having both parents as smokers carry additional risks for their offspring smoking, we have interacted fathers’ and mothers’ smoking characteristics in multilevel mixed-effects Poisson regressions with the same specifications, as in Table 4. We have not found that there is such an association. Furthermore, through interacting age and parental smoking behaviour, we did not detect any systemic and significant differences in intergenerational transmission of smoking across different age groups in either Russia or in Belarus.

## Discussion

In this article, we examined intergenerational transmission of smoking among middle-aged and older populations in Russia and Belarus using the newly available data from the PrivMort retrospective cohort study. This data set is a unique source for understanding the association of parental smoking behaviour on their offspring’s tobacco use in post-communist countries. The validity of our analysis is strengthened by the fact that the prevalence of smoking in our sample for men, 53.3 and 51.9%, respectively, in Russia and Belarus, comes close to the latest available estimates for adult populations derived from the Russia Longitudinal Monitoring Survey of RLMS-HSE for 2014 (49.0%) (Quirmbach and Gerry 2016) and the Health in Times of Transition Study (HITT) for Belarus in 2010 (42.8%) (Roberts et al. 2012). The main reason why the prevalence of current smoking among women in our data set is lower than that reported in the above-mentioned nationally representative surveys (9.4 vs. 14.4% for Russia and 8.3 vs. 13.2% for Belarus) is that the PrivMort does not include groups aged 41 and below who have the highest prevalence of smoking.

Adjusting for social origin, socio-demographic, and socio-economic variables, we find that paternal smoking significantly increases the chances of smoking among sons, while having a regularly smoking mother significantly increases the chances of smoking among daughters. In absolute terms, this equates to about 9–12 percentage points higher prevalence of smoking in Russia and Belarus among women with smoking mothers in comparison to women with never smoking mothers, which is remarkable, considering that in our sample of middle-aged and older women, the rate of smoking in Russia and Belarus is less than 10%. These results are in line both with psycho-analytic theory (Boyd 1989) which claims that daughters tend to unconsciously internalise maternal values and behaviours as well as with social learning theory (Bandura 1977) that emphasises principles of intergenerational modelling and suggests that girls are consistently and positively reinforced when they learn to be like their mothers and imitate maternal behaviour. Daughters also tend to spend significantly more time with their mothers than sons do (Kislitsyna et al. 2010). Our results complement the earlier findings on the parental transmission of smoking that do not usually emphasise the varying roles of paternal and maternal tobacco use in middle-aged and older sons and daughters smoking behaviour (e.g., Brenner and Scharrer 1996; Bricker et al. 2006; Chassin et al. 1998; Kandel and Wu 1995; Melchior et al. 2010).

Our study has a number of limitations. Due to the survey design, we do not have information on smoking for those parents who died before 1982. Therefore, information on maternal smoking had to be imputed for 10 and 7% of male and 12 and 7% of female respondents, respectively, in Russia and Belarus. Furthermore, the data set is not a nationally representative survey of Russia and Belarus and the findings cannot be generalised to these countries’ entire populations. Another limitation of this study is recall bias and measurement error that can stem from asking questions about circumstances and events related to respondents’ parents and their childhood. Such misclassification can lead to misestimating of the strengths of associations between parental and offspring smoking. The design of the questionnaire we use, however, mitigates this limitation by introducing auxiliary sentences and incorporating active visualisation memory cards that can assist people in remembering various characteristics of their parents’ and their own childhood more easily.

Our findings not only contribute to the existing scholarship on intergenerational transmission of socio-economic disadvantage in post-communist contexts (Gugushvili 2015, 2016, 2017a, b), but are also relevant in interpreting trends and patterns of smoking among middle-aged and older Russian and Belarusian men and women, with implications for subsequent generations, an issue of importance given increasing smoking among women. Of course, we cannot assume that the strength of intergenerational transmission of smoking will be the same in the future, given the many other factors involved such as shrinking gender differences in smoking. Nonetheless, if parents are aware of the implications of smoking and how much they can influence their sons and daughters’ propensity to smoke even in the later stages of their life course, some might have an extra reason not to start smoking or to quit after smoking initiation (Bricker et al. 2006; Roberts et al. 2013).

However, the main implication of our results for research on prevention and control of tobacco use is that intergenerational transmission of smoking does not seem to be a methodological artefact of inadequately accounting for social origin variables and the many potentially confounding factors throughout individuals’ lives. Parental influence on smoking is a significant factor not only for adolescents but also for middle-aged and older populations in the considered post-communist countries. Therefore, to understand thoroughly the confounding factors of smoking in the region and, arguably, beyond, the existing longitudinal and cross-sectional surveys, such as Global Adult Tobacco Survey (GATS) and RLMS-HSE, should explicitly enquire into smoking behaviours of respondents’ parents. Intergenerational influences on smoking should also be taken into account of in studies seeking to monitor rates of smoking and the impact of tobacco control programmes.
